# Prevalence and Factors of Anxiety During the Coronavirus-2019 Pandemic Among Teachers in Saudi Arabia

**DOI:** 10.3389/fpubh.2022.827238

**Published:** 2022-03-21

**Authors:** Riyadh A. Alhazmi, Sultan Alghadeer, Mohamed N. Al-Arifi, Asma A. Alamer, Abdullah M. Mubarak, Abdulrahman Alwhaibi, Raghad Alfayez, Sara Alsubaie

**Affiliations:** ^1^Department of Emergency Medical Services, Prince Sultan Bin Abdulaziz College for Emergency Medical Services, King Saud University, Riyadh, Saudi Arabia; ^2^Department of Clinical Pharmacy, College of Pharmacy – King Saud University, Riyadh, Saudi Arabia; ^3^Department of Curricula and Teaching Methods, College of Education, King Faisal University, Al-Ahsa, Saudi Arabia; ^4^Department of Basic Science, Prince Sultan Bin Abdulaziz College for Emergency Medical Services, King Saud University, Riyadh, Saudi Arabia

**Keywords:** COVID-19, pandemic, anxiety, education, occupational health, educator, teacher, mental

## Abstract

**Background:**

Teachers play a central role in successful education. Due to the COVID-19 pandemic, regular in-person attendance in classes at all levels of education has been disrupted for more than 1 year in many countries. These lockdowns, which include the discontinuation of in person learning at schools and universities has presented a significant challenge for teachers to adapt to online teaching. Given this rapid format change, occupational anxiety levels among educators has increased.

**Objective:**

The primary objective of this study was to assess the prevalence of anxiety among teachers in Saudi Arabia. A secondary objective was to explore characteristics of teachers associated with the level of anxiety level during the period of lockdown.

**Methods:**

An anonymous, online cross-sectional study was carried for 3 months (February 2021 through April 2021). The questionnaire consisted of four sections and included the Generalized Anxiety Disorder instrument (GAD-7). Chi-square tests were completed for categorical comparisons while binary logistic regressions were used for associative relationship exploration. The IRB at King Saudi University Medical City, Saudi Arabia approved this study.

**Results:**

A total of 742 respondents completed the survey yielding an anxiety prevalence of 58.2 % among teachers. Medium degree of statistically significant differences identified as marital status (*p* = 0.046). women had higher anxiety (65.3%) than men (34.7%) but gender with anxiety was low degree of statistical significance compared with non-anxiety status (*p* = 0.697). The odds of anxiety among middle teachers was twice (OR = 2.01) as high as the odds of anxiety among other levels of teacher (*p* = 0.01, 95% CI 0.94–4.26).

**Conclusions:**

This study identified that many teachers experienced anxiety during the lockdown, especially women and middle school teachers. Future studies should identify contributing factors to estimate the magnitude of the exposure to anxiety between different types of teachers to help establish better preventive measures based on the workplace environment.

## Introduction

With advances in education and curriculum, the teacher still plays a central role in a successful educational experience. Formal education is an integrated system, relying upon systematic and structural approaches provided in educational facilities (1). Through teachers, the learning process of formal education is intended to provide students with essential knowledge and skills required to achieve their desired goals. Therefore, regular in-person attendance for teachers and students is mandatory in most educational environments (2). However, many of the standard approaches have been suspended due to the COVID-19 pandemic.

While the disease is theorized to have emerged in Wuhan, China in December 2019 in <3 months the World Health Organization (WHO) declared COVID-19 a global pandemic (3–5). Shortly thereafter, the WHO announced comprehensive recommendations and preventive measures to reduce transmission and decrease the rate of new infections (5). The rapid onset and global spread of COVID-19 is not only one of the most critical public health emergencies in modern times, but the cascading effects on the health and wellbeing of persons is concurrently impacted across many areas of like. As a result of the pandemic, the changing of many aspects of life combined with a constant concern of transmission of infection, has increased anxiety worldwide (6).

Many countries adhered to WHO's recommendations including travel and work restrictions and educational institutions lockdown (5). In addition to their regular workload, teachers experienced a significant shift to online learning in many countries. The additional burdens of developing and deploying new teaching methods while potentially being exposed to a novel pathogen in-person led to a cumulative impact on the stress and anxiety among teachers (7, 8).

Temporary feelings of being anxious or tense will impact persons differently than chronic anxiety as these tend to be acute episodes. However, an event like a pandemic, can lead to extended anxiety, whether a formal anxiety disorder or chronic feelings of anxiousness, these episodes ng can have an adverse effect on one's quality of life as well as mental and physical health (9). Extended periods of anxiety and anxiety disorders can lead to other serious medical conditions such as heart diseases and cancer (10, 11). Otherwise healthy people experiencing high levels of stress can eventually develop health anxiety (12) which will cause people to suffer more, and have influence their thinking and decision-making processes in day to day life (13).

One study showed increased anxiety levels among teachers during the COVID-19 pandemic (14). High school teachers were found to develop an anxiety disorder more than teachers in other stages of education (15). Studies have assessed the incidence of anxiety among different occupations, finding teachers among the most impacted among occupations (16–23). Specifically, female teachers had higher levels of anxiety than male teachers. On the other hand, previous studies have not addressed an association between married teachers and anxiety level, as may be a step in realizing a contributing factor in anxiety level. Although, studies shown positive association between media exposure and anxiety level (24). More precisely, several studies showed exposure to different types of media information such as a twitter, TV news and other sources can a play an important role in anxiety level (25–27). Similarly, social media greatly impacted the level of anxiety during the period of COVID-19 (16, 26, 28). Evidence suggests that sharing concerns by social media improved people's mental condition for the period of COVID-19 crisis (29).

In Saudi Arabia, the first confirmed case was on March 2, 2020, resulting in implementing preventive measures in a line with the WHO guidelines (30). Our restrictive lockdowns included the closure of schools and universities was created a huge challenge to teachers to find ways to adapt to online teaching method. The stress of the pandemic and its impact on daily life can has significant effects on occupational health. Teachers around the world have been impacted by the lockdowns, however, there is very limited research examining teacher's mental health during COVID-19 in many nations, including Saudi Arabia. The primary objective of this study was to assess the prevalence of a state of anxiety among teachers in Saudi Arabia. A secondary objective was to explore characteristics of Saudi teachers and their association with anxiety level during the period of lockdown.

## Methods

### Study Design and Population

A cross sectional study was completed using an anonymous online survey to assess the anxiety among Saudis' teachers during the period of COVID-19 pandemic. An invitational email was sent to educational regions in Saudi Arabia, in turn they distributed the survey link to comprehensive list of public and private education from primary through college teachers, instructors and faculty members. Study instructions and an electronic cover letter were shown at the beginning of the survey. The survey was conducted for 3 months (February 2021 through April 2021) to assess the prevalence of anxiety and its contributing factors. It was estimated that the total number of teachers in public schools and universities in Saudi Arabia is around 577,700 teachers according to the latest annual report of the Saudi Arabia Monetary Agency for population and workforce (31). Based on this number, the minimum sample size was calculated, with 95% confidence level and 5% margin of error, to be 384 participants. The institutional research board (IRB) at King Saud University Medical City approved the conduction of study (No. E-21-5914).

### Survey Instrument and Data Analysis

The questionnaire consists of three sections, including demographics (i.e., age, gender, educational level, type of school, school location, and income), in addition to sources for gaining information about COVID-19. The second section included the behavioral status and commitment of teachers to the health policy restricted regulations toward COVID-19. All behavioral questions such as wearing mask, increased hand washing, social distancing, and limited family gatherings were categorized into three levels: high (if all answers were correct), moderate (with some correct answers), and low/none (with incorrect answers for all questions). The final section was the Generalized Anxiety Disorder instrument (GAD-7) of an Arabic version and was used after the permission of author was obtained.

Cronbach's alpha has been calculated with 0.817 (α > 0.7) of the behavioral status and GAD-7 all together and reported in additional file (Supplementary Material 3). A comparison of the instruments found the Cronbach's alpha was 0.763 (32). The Inter-Item Correlation Matrix showed there is sufficiently weak correlation between the independent variables (<0.7) (Supplementary Material 3). It was assessed by scores of 0, 1, 2, and 3 to answers options of (never, several days, more than half the days and nearly every day). The total score ranged from 0 to 4 indicated no anxiety, scores of 5–9 indicated mild anxiety, scores of 10 to 14 showed moderate anxiety and scores of 15–21 showed severe anxiety.

Categorical data were shown as frequency, mutually exclusive and had expected count <5 with 20.14 of the minimum expected count, compared using chi-squared test. Categorical data were shown as frequency including dichotomous outcome with, dichotomous nominal and ordinal independent variables. Spearman's rho was performed to test outliers and correlation (Supplementary Material 2). Consequently, the data successfully met the assumptions that are required for Binary logistic regression model used to obtain odds ratio (OR) and their associated 95% confidence interval (CI).

The degree of statistical significance was set based on near or far from a *P*-value of = 0.05 with very high, high, medium, low, and very low significance to a *P*-value of = 0.05. Analysis was performed using Statistical Package for the Social Sciences 24 (IBM-SPSS-24). The datasets generated during and/or analyzed during the current study are available from the corresponding author on reasonable request.

## Results

**Figure 1:**
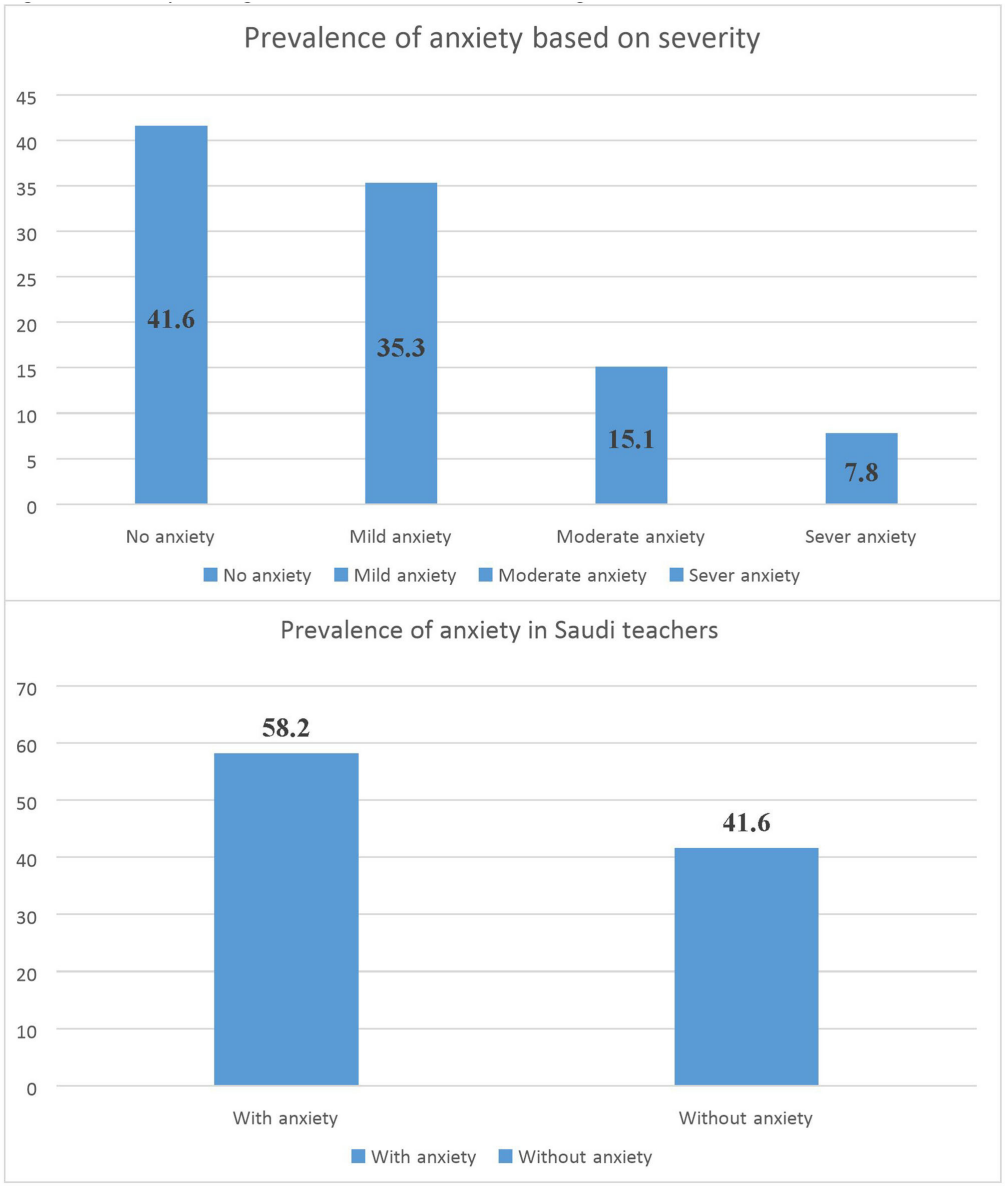
Anxiety among teachers in Saudi Arabia during COVID-19 pandemic.

A total of 742 respondents completed the survey. Most respondents (64.6%) were women. According to an Arabic version of GAD-7 survey, the prevalence of anxiety in Saudi teachers was 58.2%, showed by Figure 1. Of these 742 respondents, 42, 35, 15, and 8%. Had no, mild, moderate and severe anxiety, respectively, described in Figure 1.

Table 1 presents the features of respondents by anxiety vs. non-anxiety status with the only medium degree of statistically significant differences identified as marital status (*p* = 0.046). Our results showed that women had higher anxiety (65.3%) than men (34.7%) but gender with anxiety was low degree of statistical significance compared with non-anxiety status (*p* = 0.697). In addition, fears teaching online and getting infected were very high degree of statistical significance *P* = 0.001 with OR and 95%CI of 1.936 (1.319–2.841) and 1.739 (1.246–2.426), respectively. However, the behavior status was very low degree of statistical significance.

**Table 1:** Demographics of the teachers based on their anxiety status.

**Demographics**	**All respondent** **(*n* = 742)**	**With anxiety** **(*n*= 432)**	**Without anxiety** **(*n* = 310)**	*P*-value^*^
**Sex**				0.697
Male	263 (35.4%)	150 (34.7%)	112 (36.2%)	
Female	479 (64.6%)	282 (65.3%)	197 (63.8%)	
**Marital status**				0.046
Married	426 (57.4%)	233 (54.2%)	192 (63.4%)	
Single	271 (36.5%)	172 (40.1%)	99 (32.7%)	
Divorced	36 (4.9%)	24 (5.6%)	12 (9.0%)	
**Type of teachers**				0.051
Primary teachers	206 (27.8%)	108 (25%)	98 (31.7%)	
Secondary teachers	88 (11.9%)	61 (14.1%)	27 (8.7%)	
High teachers	239 (32.2%)	114 (33.3%)	95 (30.7%)	
University teachers	209 (28.2%)	119 (27.55%)	89 (28.8%)	
**Information resources**
Internet	281 (37.9%)	147 (34.3)	133 (43.2)	
Friends	2.8 (21.0)	13 (3.0)	8 (2.6)	0.061
Social media	329 (44.3)	208 (48.6)	121 (39.3)	
TV	106 (14.3)	60 (14.0)	46 (14.9)	
**Teach online**				0.003
Yes	217 (29.2)	145 (33.8)	72 (23.8)	
No	516 (69.5)	284 (66.2)	231 (76.2)	
**COVID 19 infected**
Yes	346 (46.6)	223 (51.6)	123 (39.8)	0.002
No	396 (53.4)	209 (48.4)	185 (60.2)	

* *P-value is calculated by Chi Square test*.

Participant's responses were explored to look for an association using binary logistic regression with binary outcome of anxiety and without anxiety as shown in Table 2. Male gender was found to have very low degree of a statistically significantly association (*p* = 0.36, OR 0.830, 95% CI 0.556–1.240). However, the odds of anxiety among middle teachers was twice (OR = 2.01) as high as the odds of anxiety among other levels of teacher (*p* = 0.01, 95% CI 0.94–4.26). Furthermore, teachers who used social media as source of pandemic information had increased 1.6 times the odds of anxiety compared to persons not reporting pandemic related information from social media (95% CI, 1.08–2.3) with *p* = 0.02.

**Table 2:** Association between participants responses with presence of anxiety.

**Demographics**	**OR (95% CI)**	*p*-value^*^
**Gender**
Sex	Reference	-
Male	0.830 (0.556–1.240)	0.363
**Marital status**
Married	Reference	-
Single	1.334 (0.835–2.130)	0.228
Divorced	1.543 (0.684–3.485)	0.296
**Education level**
Primary/secondary school	0.957 (0.162–5.638)	0.961
High school	2.005 (0.944–4.262)	0.070
University	1.352 (0.767–2.384)	0.307
High education	Reference	-
**Type of teachers**
Primary teachers	Reference	-
Secondary teachers	2.091 (2.091–1.169)	0.013
High teachers	1.128 (0.728–1.749)	0.590
University teachers	1.123 (0.664–1.901)	0.665
**Information resources**
Internet	Reference	-
Friends	2.068 (0.713–5.998)	0.181
Social media	1.557 (1.083–2.237)	0.017
TV	1.315 (0.789–2.189)	0.293
**Teach online**
No	Reference	-
Yes	1.936 (1.319–2.841)	0.001
**COVID 19 infected**
No	Reference	-
Yes	1.739 (1.246–2.426)	0.001

*OR, odds ratio; CI, confidence interval.*

* *Significant result at a = 0.05*.

## Discussion

Education is a fundamental institution for development of social and cultural aspects in every country. Schools serve not only as learning centers but as centers for development. The COVID-19 pandemic response resulted in schools and universities in most countries including Saudi Arabia being closed and moved to online educational methods (5). The rapid proliferation of online education through various digital platforms not only impacts a person's teaching skills but also their mental health.

In this study of 742 teachers completing an online cross-sectional survey, the majority of teachers (58.2%, *n* = 433) reported increased anxiety during the lockdown, with 35.3% reporting mild anxiety. While globally there is limited research in this area, these findings align with previously conducted research confirming teacher anxiety during lockdown (14). However, we believe there may be significant underreporting of anxiety due in part to the time of this study and that the teachers may be underestimating the situation. The nature of teaching requires continuous work and daily preparations to carry out the educational objectives. This effort, by its nature, is a source of increasing stress and anxiety level among teachers (7, 8). Nevertheless, the anxiety felt by the teachers during the COVID-19 pandemic has been higher than prior to the lockdowns (14). This is likely due to restricted social movements and consistent health anxiety and concerns about the pandemic (33). In addition, utilizing online teaching method involves a high level of anxiety among teachers (14).

Teachers serve different stages of education such as primary, middle, high school and… etc. Therefore, they face varying levels of anxiety and stress. In COVID-19 pandemic, previous research found high school teachers confronted an increased level of anxiety and stress compared to other stages of education (15). In our study, the findings showed an association between the types of teacher stage and anxiety, in which middle teachers were highly associated with anxiety level during the lockdown. In contrast, Ozamiz-Etxebarria et al. found primary teachers showed a high level of anxiety during lockdown (14). These results indicate teachers are exposed to a great amount of anxiety and stress depends on the stage of education. Our result showed ages group of middle education (13–15 years) could cause more stress and pressure to the teachers. This could be students at these ages want to be more independent and give physiological changes can be more irritable, distant, and disobedient (34). Consequently, it can be a source of stress and conflict for teachers working in the middle school education. Not to mention, online teaching requires more attention from teachers which increases challenges in completing all the new requirements in a timely manner. The result indicates a need for further research to identify factors that might be a cause of anxiety for middle teachers during COVID-19 outbreak and lockdown.

The sex differences in anxiety levels have been the subject of numerous studies with women more likely to report anxiety during the current pandemic (16–23). In our study, the results found similar degree of evidence between anxiety and sex. However, our study showed marital status to be a factor at increased level of anxiety. This suggests married teachers are more likely to display higher levels of anxiety than their single counterpart. The pandemic could have an amplifying effect to anxiety especially for teachers with children because they have to adapt to new teaching strategies using different medium of instruction along with childcare and household responsibilities.

In our study, there is also a positive association between anxiety and social media exposure compared to other information resources. Recent research has recognized a positive association between media exposure and anxiety before and during the current pandemic (16, 24, 26, 28). In addition, studies conducted in Saudi Arabia (25), UK (26), and China (27) showed the effects of different types of media sources on person's anxiety level. A consensus was reached that social media consumption is linked to higher levels of anxiety compared to other media platforms. Similarly, our findings suggest that teachers who receive their information about the pandemic from social platforms are also more likely to have anxiety. However, it is unclear whether persons with anxiety tend to use social medial platforms to seek information or that social media consumption aggravates mental health issues. Therefore, establishing any cause and effect relation could be misleading. Social media could have positive effect on mental health such as providing social support through this difficult period (29). On the other hand, misinformation along with rumors are easily disseminated through social media platforms in comparison to traditional platforms where information is verified and controlled. Future research should evaluate the difference between types of information shown in different types of media resources as well as how fast it can impact teachers' perspective.

## Limitations

In our study, numerous limitations need to be recognized. First, our findings are not generalizable to the entire population because of the cross-sectional nature of the research. Second, the study did not cover age groups and years of experience of teachers. Varying ages and years of experience might be a factor of negatively or positively increasing a level of anxiety in the COVID-19 pandemic. Third, there is also the possibility of selection bias since the research was performed with an online questionnaire. Teachers who are unable or unwilling to use smartphones or email could not participate in the study. Fourth, the study did not include a section related to teachers with pre-existing anxiety disorders such as panic attack, social health anxiety, social phobia, or generalized anxiety disorder (GAD) that can be associated with teachers. Future research should consider pre-existing anxiety disorders when carrying out teachers' mental health study. Last, the study showed marital status is associated with level of anxiety, and yet, the study did not address the number of children one's have and their possible effect on anxiety.

## Conclusion

This study identified that many teachers experienced anxiety during the lockdown. We found that most teachers (58.2%, *n* = 432.5) reported anxiety during the lockdown especially women and middle school teachers. Future studies should identify contributing factors to estimate the magnitude of the exposure to anxiety between different types of teachers to help establish better preventive measures based on the workplace environment. In addition, our study showed a positive association between anxiety and social media exposure compared to other information resources. Future research should evaluate the difference between types of information shown in different types of media resources as well as how fast it can impact teachers' perspective.

## Data Availability Statement

The raw data supporting the conclusions of this article will be made available by the authors, without undue reservation.

## Ethics Statement

The studies involving human participants were reviewed and approved by IRB approval letter. The patients/participants provided their written informed consent to participate in this study.

## Author Contributions

RAA, SAlg, and MA-A provided the main framework and identified main factors and materials. AAla, AAlw, and RA collaborated in identifying appropriate references and collaborated in writing the manuscript. SAls and SAlg were involved in data collection and cleaning data. AM and MA-A were involved in data analysis, interpreted the results, and collaborated in writing the manuscript. SAlg, RAA, and AAla collaborated in writing and editing the paper. All authors read and agreed to the published version of the manuscript.

## Conflict of Interest

The authors declare that the research was conducted in the absence of any commercial or financial relationships that could be construed as a potential conflict of interest.

## Publisher's Note

All claims expressed in this article are solely those of the authors and do not necessarily represent those of their affiliated organizations, or those of the publisher, the editors and the reviewers. Any product that may be evaluated in this article, or claim that may be made by its manufacturer, is not guaranteed or endorsed by the publisher.
