# Supplementary material for: Prevalence and Factors of Anxiety During the Coronavirus-2019 Pandemic Among Teachers in Saudi Arabia
Source: Front Public Health. 2022 Mar 21;10:827238. doi: 10.3389/fpubh.2022.827238 (PMC8978600; doi:10.3389/fpubh.2022.827238)
Supplement: Supplementary file 1 [file Data_Sheet_2.pdf]

Binary logistic regression model

Spearman's rho **for Correlations**

		**gender**	**Material Status**	**Education**	**Location**	**Behaviour status**
gender	Correlation Coefficient	1.000	.300**	-.029-	.207**	-.120-**
	Sig. (2-tailed)	.	.000	.432	.000	.001
	N	742	733	741	742	741
Material _Status	Correlation Coefficient	.300**	1.000	-.237-**	.325**	-.130-**
	Sig. (2-tailed)	.000	.	.000	.000	.000
	N	733	733	732	733	732
Education	Correlation Coefficient	-.029-	-.237-**	1.000	.155**	.143**
	Sig. (2-tailed)	.432	.000	.	.000	.000
location	Correlation Coefficient	.207**	.325**	.155**	1.000*	-.067-
	Sig. (2-tailed)	.000	.000	.000	.	.068
Behaviour status	Correlation Coefficient	-.120-**	-.130-**	.143**	-.067-	1.000
	Sig. (2-tailed)	.001	.000	.000	.068	.
	N	741	732	740	741	741

**. Correlation is significant at the 0.01 level (2-tailed).

*. Correlation is significant at the 0.05 level (2-tailed).
