# Supplementary material for: Prevalence and Factors of Anxiety During the Coronavirus-2019 Pandemic Among Teachers in Saudi Arabia
Source: Front Public Health. 2022 Mar 21;10:827238. doi: 10.3389/fpubh.2022.827238 (PMC8978600; doi:10.3389/fpubh.2022.827238)
Supplement: Supplementary file 2 [file Data_Sheet_3.pdf]

Cronbach's alphas

---

1. **The behavioural and commitment of teachers to the health policy restricted regulations towards COVID-19(** Cronbach's alphas with their 95% confidence interval and Inter-Item Correlation Matrix for the behavioural status section)

Item	Item Statistics			Inter-Item Correlation Matrix			
	**Mean**	**Std. Deviation**	**N**	**wearing a mask**	**Hand Washing**	**not attending family meeting**	**Not to be in crowded places**
**wearing a mask**	2.56	.644	741	1.00	.317	.149	.129
**Hand Washing**	2.42	.709	741	.317	1.00	.314	.254
**not attending family meeting**	2.21	.761	741	.149	.314	1.00	.591
**Not to be in crowded places**	2.38	.73	741	.129	.254	.591	1.00
	**Mean**	2.39					
	**Variance**	.021					

Intraclass Correlation Coefficient

	Intraclass Correlation[b]	95% Confidence Interval		F Test with True Value 0			
		**Lower Bound**	**Upper Bound**	**Value**	**df1**	**df2**	**Sig**
**Single Measures**	.298[a]	.260	.338	2.701	740	2220	.000
**Average Measures**	.630[c]	.584	.671	2.701	740	2220	.000

Reliability Statistics

	Cronbach's Alpha	Cronbach's Alpha Based on Standardized Items	N of Items
	.630	.623	4

.

2. Generalized Anxiety Disorder (GAD) (Cronbach's alphas with their 95% confidence interval and Inter-Item Correlation Matrix for the GAD section)

Item	Item Statistics		Inter-Item Correlation Matrix						
	**Mean**	**Std. Deviation**	**Q1**	**Q2**	**Q3**	**Q4**	**Q5**	**Q6**	**Q7**
**Q1. Feeling nervous, anxious or on edge?**	1.04	.861	1.000	.678	.608	.550	.488	.589	.527
**Q2. Not being able to stop or control worrying?**	.84	.868	.678	1.000	.683	.605	.512	.564	.578
**Q3Worrying too much about different things?**	1.08	.913	.608	.683	1.000	.639	.514	.594	.580
**Q4. Trouble relaxing?**	.98	.917	.550	.605	.639	1.000	.567	.581	.545
**Q5. Being so restless that it is hard to sit still?**	.64	.868	.488	.512	.514	.567	1.000	.552	.626
**Q6. Becoming easily annoyed or irritable?**	1.00	.948	.589	.564	.594	.581	.552	1.000	.565
**Q7. Feeling afraid as if something awful might happen?**	.74	.918	.527	.578	.580	.545	.626	.565	1.000
	**Mean** 2.39								
	**Variance** .021								

Intraclass Correlation Coefficient

	Intraclass Correlation[b]	95% Confidence Interval		F Test with True Value 0			
		**Lower Bound**	**Upper Bound**	**Value**	**df1**	**df2**	**Sig**
**Single Measures**	.578[a]	.548	.607	10.576	740	4440	.000
**Average Measures**	.905[c]	.895	.915	10.576	740	4440	.000

Reliability Statistics

Cronbach's Alpha	Cronbach's Alpha Based on Standardized Items	N of Items
.905	.906	7

3. **GAD score and the behavioural and commitment of teachers to the health policy restricted regulations towards COVID-19 only (** Cronbach's alphas with their 95% confidence interval and Inter-Item Correlation Matrix for the GAD section and Behaviour status)

Item	Inter-Item Correlation Matrix										
	**wearing a mask**	**Hand Washing**	**not attending family meeting**	**Not to be in crowded places**	**Q1**	**Q2**	**Q3**	**Q4**	**Q5**	**Q6**	**Q7**
**wearing a mask**	1.000	.317	.149	.129	-.001-	.000	.017	-.006-	-.042-	.003	-.043-
**Hand Washing**	.317	1.000	.314	.254	.089	.041	.022	.044	.000	-.053-	.014
**not attending family meeting**	.149	.314	1.000	.591	.016	.076	.064	.053	.026	.040	.039
**Not to be in crowded places**	.129	.254	.591	1.000	.030	.044	.050	.053	-.007-	.019	.005
**Q1.**	-.001-	.089	.016	.030	1.000	.678	.608	.550	.488	.589	.527
**Q2.**	.000	.041	.076	.044	.678	1.000	.683	.605	.512	.564	.578
**Q3**	.017	.022	.064	.050	.608	.683	1.000	.639	.514	.594	.580
**Q4.**	-.006-	.044	.053	.053	.550	.605	.639	1.000	.567	.581	.545
**Q5.**	-.042-	.000	.026	-.007-	.488	.512	.514	.567	1.000	.552	.626
**Q6.**	.003	-.053-	.040	.019	.589	.564	.594	.581	.552	1.000	.565
**Q7.**	-.043-	.014	.039	.005	.527	.578	.580	.545	.626	.565	1.000
	**Mean** 2.39										
	**Variance** .021										

Intraclass Correlation Coefficient

	Intraclass Correlation[b]	95% Confidence Interval		F Test with True Value 0			
		Lower Bound	Upper Bound	Value	df1	df2	Sig
**Single Measures**	.289[a]	.263	.317	5.468	740	7400	.000
**Average Measures**	.817[c]	.797	.836	5.468	740	7400	.000

Reliability Statistics

Cronbach's Alpha	Cronbach's Alpha Based on Standardized Items	N of Items
.817	.797	11

Two-way mixed effects model where people effects are random and measures effects are fixed.

a. The estimator is the same, whether the interaction effect is present or not.

b. Type C intraclass correlation coefficients using a consistency definition-the between-measure variance is excluded from the denominator variance.

c. This estimate is computed assuming the interaction effect is absent, because it is not estimable otherwise.
